# Extracorporeal near-infrared–activated antimicrobial photodynamic therapy for pneumonia using an ICG–polymer formulation

**DOI:** 10.1117/1.BIOS.3.3.035002

**Published:** 2026-08-13

**Authors:** Lorraine Gabriele Fiuza, Isabelle Almeida de Lima, Fernanda Alves, Michelle Barreto Requena, Vadila Giovana Guerra, Layla Pires, Cristina Kurachi, Natalia Mayumi Inada, Vanderlei Salvador Bagnato

**Affiliations:** a University of São Paulo, São Carlos Institute of Physics, São Carlos, Brazil; b Texas A&M University, Department of Biomedical Engineering, College Station, Texas, United States; c Federal University of São Carlos, Department of Chemical Engineering, São Carlos, Brazil

**Keywords:** animal model studies, bacteria, indocyanine green, lung, near infrared, photodynamic therapy, polymers, resistance, pneumonia, Streptococcus pneumoniae

## Abstract

**Significance:**

Pneumonia remains a global health challenge, exacerbated by antimicrobial resistance, which limits the effectiveness of conventional treatments. Antimicrobial photodynamic therapy (aPDT) has emerged as a promising alternative for lower respiratory infections; however, its *in vivo* performance is hindered by lung surfactant, which traps photosensitizers and reduces their ability to reach bacterial targets.

**Aim:**

To address this limitation, recent studies demonstrated that combining indocyanine green (ICG) with the polymer Gantrez AN-139 enhances photodynamic activity even in the presence of lung surfactants.

**Approach:**

We assessed the therapeutic potential of this formulation in a murine pneumonia model using pulmonary delivery followed by 808 nm infrared irradiation.

**Results:**

Nebulization of 200  μM ICG with 4% Gantrez AN-139 and irradiation at $120\;J/{\mathrm{cm}}^{2}$ yielded the best results, reducing bacterial load by up to $3\;\log_{10}$ and improving survival to 80%, compared with 40% in controls.

**Conclusion:**

These findings validate the formulation and the procedure for further animal studies.

Statement of Discovery This work demonstrates that nebulized ICG–Gantrez AN-139, activated by extracorporeal near-infrared light, overcomes the pulmonary surfactant barrier that has limited antimicrobial photodynamic therapy in the lungs. In a murine pneumonia model, this noninvasive approach reduced bacterial burden by nearly 3 log and doubled survival from 40% to 80%. These findings support extracorporeal aPDT as a promising adjunct therapy for antibiotic-resistant respiratory infections.

## 1 Introduction

Pathogens such as fungi, viruses, and bacteria cause respiratory tract infections, with pneumonia being the most prevalent and a leading cause of global mortality and morbidity. A systematic review by Bender et al., covering the period from 1990 to 2021, identified *Streptococcus pneumoniae* as the primary etiological agent, accounting for the largest number of lower respiratory infection episodes and deaths, with approximately 97.9 million cases and 505,000 deaths worldwide.^1^^–^^6^ Bacterial pneumonia remains particularly severe among the elderly and children under five as it induces alveolar inflammation, fluid accumulation, and compromised respiratory function. Considering the growing antimicrobial resistance and the declining efficacy of conventional antibiotics, there is an urgent demand for alternative therapeutic strategies. In this context, antimicrobial photodynamic therapy (aPDT) has emerged as a promising and innovative approach. This technique combines a photosensitizer (PS), light, and molecular oxygen to generate reactive oxygen species that damage microbial cell walls and membranes, thereby inactivating pathogens. Beyond its broad-spectrum antimicrobial effect, aPDT offers several advantages, including a low potential for resistance development, selective and localized action, minimal toxicity, and a noninvasive treatment profile, making it an attractive alternative to conventional antimicrobial therapies.^1^^,^^7^^–^^10^

In the literature, although numerous studies have demonstrated the *in vitro* efficacy of aPDT, few have reported its application for the treatment of respiratory infections in animal models. When dealing with the respiratory system, several considerations are essential, including light penetration. Therefore, selecting a near-infrared excitable photosensitizer and light wavelength that enable greater penetration into the lungs under external irradiation is crucial. In this context, our research group has systematically investigated the use of aPDT for pneumonia treatment for over a decade, with particular focus on infections caused by *S. pneumoniae*. These studies have employed indocyanine green (ICG), a synthetic tricarbocyanine dye characterized by low toxicity and an absorption peak between 780 and 810 nm in aqueous solution. ^11^^–^^19^ Approved by the FDA in 1959, ICG has been widely employed as a fluorescent diagnostic agent in various medical applications and has also attracted attention as a PS. *In vitro* studies have confirmed its suitability, and its efficacy has been demonstrated against major pneumonia-causing pathogens, including *S. pneumoniae*, *Staphylococcus aureus*, *Pseudomonas aeruginosa*, and *Klebsiella pneumoniae*.^12^^,^^20^^–^^22^ In a murine model study conducted by Kassab et al.,^15^ pulmonary administration of ICG (100  μM) followed by photoactivation ($120\;\mathrm{mW}/{\mathrm{cm}}^{2}$, 808 nm) was validated, demonstrating preservation of healthy tissues. Furthermore, *in vitro* studies achieved bacterial reductions of $∼4\;\log_{10}$ in *S. aureus* under conditions exhibiting minimal to no toxicity to host cells.^15^ Tovar et al. employed an *ex vivo* porcine model and confirmed the feasibility of ICG (10  μM) activation through external irradiation with laser panels ($77.8\;\mathrm{mW}/{\mathrm{cm}}^{2}$, 808 nm).^16^ Nonetheless, despite the proven applicability of light exposure and PS administration in animal models, some barriers still limit microbial photoinactivation within the alveoli, such as the presence of lung surfactant (LS).^18^

LS is a complex mixture of lipids and proteins secreted by type II pneumocytes and is essential for reducing alveolar surface tension, enabling gas exchange, and contributing to pulmonary immune defense;^23^^–^^25^ however, it can impair aPDT by trapping PS through electrostatic interactions with phospholipid head groups, limiting their mobility and reducing therapeutic efficacy.^18^ To overcome this limitation, the Gantrez AN-139 copolymer has been proposed as a drug delivery system due to its biocompatibility, mucoadhesive properties, and ability to protect and stabilize bioactive molecules while enabling controlled release at the target site.^26^^–^^29^ Supporting this approach, Lima et al. (2024) demonstrated that Gantrez AN-139 carrying ICG achieved a $6.31\;\log_{10}$ reduction in *S. pneumoniae in vitro*, even in the presence of commercial LS, while also showing biocompatibility with human lung cells, highlighting its potential for pulmonary drug delivery.^19^

Building on these findings, the present study aimed to validate this protocol in a murine model to assess its effectiveness in treating pneumococcal pneumonia. By establishing a proof of concept, this research aims to develop innovative therapeutic strategies to overcome the challenges posed by antimicrobial resistance.

## 2 Materials and Methods

### 2.1 Preparation of the ICG-Gantrez Formulation

A stock solution of 30% Gantrez AN-139™ (Ashland, Wilmington, Delaware, United States) was prepared as described in the literature.^30^^,^^31^ In summary, polymer powder was diluted in Milli-Q water and heated at 95°C for 4 to 5 h to induce gel formation by hydrolysis of the copolymer’s anhydride into its corresponding acid. For the experiments, a 4% (w/v) Gantrez working solution was prepared and mixed with different concentrations of ICG. The mixture was then centrifuged at 3500 rpm for 10 min to ensure complete homogenization and removal of bubbles. For tests using only the polymer, the volume allocated for ICG was replaced with Milli-Q water, following the same preparation procedure.

### 2.2 Bacteria and Animal Models

This study was approved by the Animal Ethics Committee of the São Carlos Institute of Physics, University of São Paulo (Approval No. 6505060323). All experiments were conducted in accordance with ARRIVE guidelines and the National Council for the Control of Animal Experimentation (CONCEA). Female BALB/c mice, aged 8 weeks and weighing ∼25  g, were used, considering three to five animals per group.

*S. pneumoniae* (ATCC^®^ 49619™) was stored in Tryptic Soy Broth (TSB) with 15% glycerol at −80°C. The inoculum was prepared by adding 200  μL of the bacteria to 10 mL of brain heart infusion and incubating it in a 5% ${\mathrm{CO}}_{2}$ atmosphere at 37°C until the optical density (OD 600 nm) reached 0.2. The bacteria were then centrifuged, resuspended in phosphate-buffered saline (PBS), and prepared at a concentration of $10^{7}\;\mathrm{CFU}/\mathrm{mL}$.

Before bacterial administration, cohorts of female Balb/c mice (n=3 to 5) were immunosuppressed with two intraperitoneal injections of cyclophosphamide. An initial dose of 150  mg/kg was administered, followed by a second dose of 100  mg/kg 72 h later. Twenty-four hours after immunosuppression, the mice were infected via nasal instillation with 15  μL of *S. pneumoniae* per nostril. In all cases, treatment was applied 24 h after the disease installation.

### 2.3 Antimicrobial Photodynamic Therapy (aPDT) Protocol

For the proposed treatment, two delivery methods were tested: nebulization and intratracheal instillation. Nebulization was delivered through a face mask coupled to an ultrasonic nebulizer (Omron, Brazil). The instillation was performed by inserting a catheter into the trachea and administering 10  μL of the formulation every 2 min, for a total of 40  μL. All parameters employed are shown in Table 1.

**Table 1:** Treatment groups and aPDT protocols.

Groups	Irradiation time (min)	Fluence ($J/{\mathrm{cm}}^{2}$)	Number of treatments	ICG concentration (μM)	Gantrez AN-139 concentration (%)
**Control (infection)**	0	0	0	0	0
**Control (** GantrezAN−139+light **)**	16	120	1	0	4
**aPDT-40**	6	40	1	200	4
aPDT−40+40	6	40	2	200	4
**aPDT-60**	8	60	1	200	4
**aPDT-120**	16	120	1	200	4
aPDT−120+120	16	120	2	200	4
**aPDT-200**	20	200	1	200	4

**Fig. 1:**
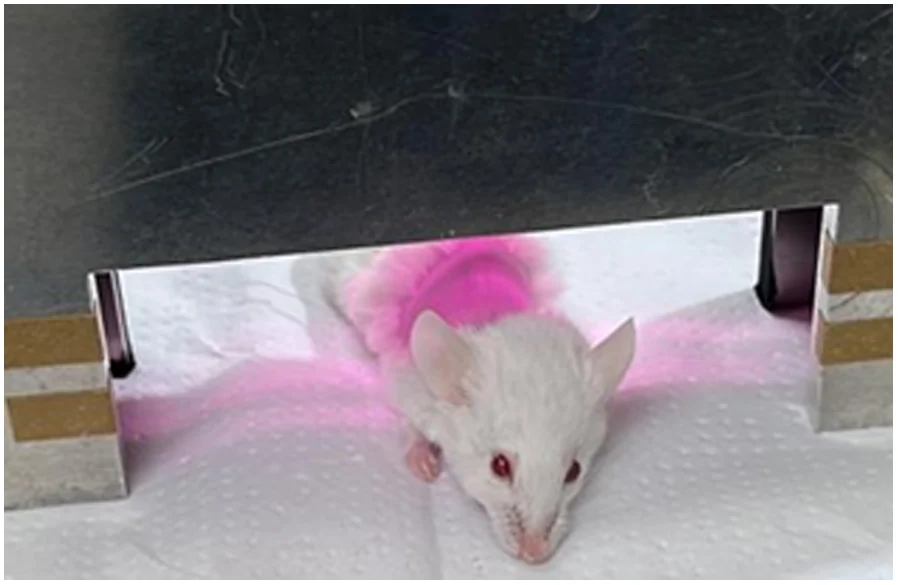
Experimental setup used for the external irradiation of the animals.

With the laser emitters configured to form a hemispherical arrangement around the animal, the system produces a light spot of $∼6\;{\mathrm{cm}}^{2}$ incident on the dorsal region, which penetrates the lungs. The treatment used a light source comprising 18 diode lasers emitting at 808 nm, arranged in a matrix to ensure uniform irradiation from all angles (Fig. 1). The irradiance at the treatment surface was measured daily with a calibrated power meter (nominal value: $120\;\mathrm{mW}/{\mathrm{cm}}^{2}$, variation: $\pm 10\;\mathrm{mW}/{\mathrm{cm}}^{2}$), and irradiation times were calculated accordingly to deliver the target fluence for each experimental group, with exposure times rounded to the nearest minute. The ICG-Gantrez formulation (nebulization) and light were delivered simultaneously. In the double-treatment (aPDT-40+40 and aPDT-120+120) groups, the treatments were applied 1 h apart.

### 2.4 Analysis of Treatment Response

#### 2.4.1 Bacterial recovery of the lungs

Two recovery methods were used: bronchoalveolar lavage (BAL) and tissue maceration. For the BAL, the mice were anesthetized with xylazine and ketamine at 10 and 100  mg/kg, respectively. A 20-gauge catheter was inserted into the trachea. The BAL was then collected by injecting 1 mL of PBS into the catheter and aspirating the fluid back through the catheter. The sample was then centrifuged at 3000 rpm for 15 min, and the supernatant was discarded. The recovered cells were resuspended in 300  μL of PBS. The lungs were then macerated in 1.0 mL of PBS. Both the BAL and the macerates were diluted and plated on blood agar plates and incubated at 37°C for 18 to 24 h. Subsequently, colony-forming units were counted.

#### 2.4.2 Survival rate

The optimized aPDT protocol was repeated in a mouse cohort (n=5) to assess whether the treatment could improve survival. The animals were monitored daily for up to 21 days. Humane endpoints included weight loss of more than 20%, shortness of breath, hunched posture, reduced movement, and ruffled fur coat.

### 2.5 Statistical Analysis

A two-way ANOVA, followed by the appropriate post hoc multiple comparisons test (GraphPad Prism 8.0), was used to assess differences between treated and untreated mice and between recovery methods (BAL versus tissue homogenization). Statistical significance was set at p<0.05. The sample size (n=3 to 5 animals per group) was determined in accordance with the principles of the 3Rs (replacement, reduction, and refinement) and the recommendations of our Institutional Animal Care and Use Ethics Committee (Approval No. 6505060323). In addition, group sizes were based on previously published studies from our group using the same murine model and comparable sample sizes.^15^

## 3 Results

### 3.1 Antimicrobial Photodynamic Therapy

The nebulized ICG-Gantrez solution was characterized using light scattering with a Spraytec System (model RTS5134 from Malvern Instruments, Malvern, Worcestershire, United Kingdom), and the typical particle diameter was found to be around 9  μm, with a dispersion of ∼1  μm (data not shown).

#### 3.1.1 Single-treatment response

**Fig. 2:**
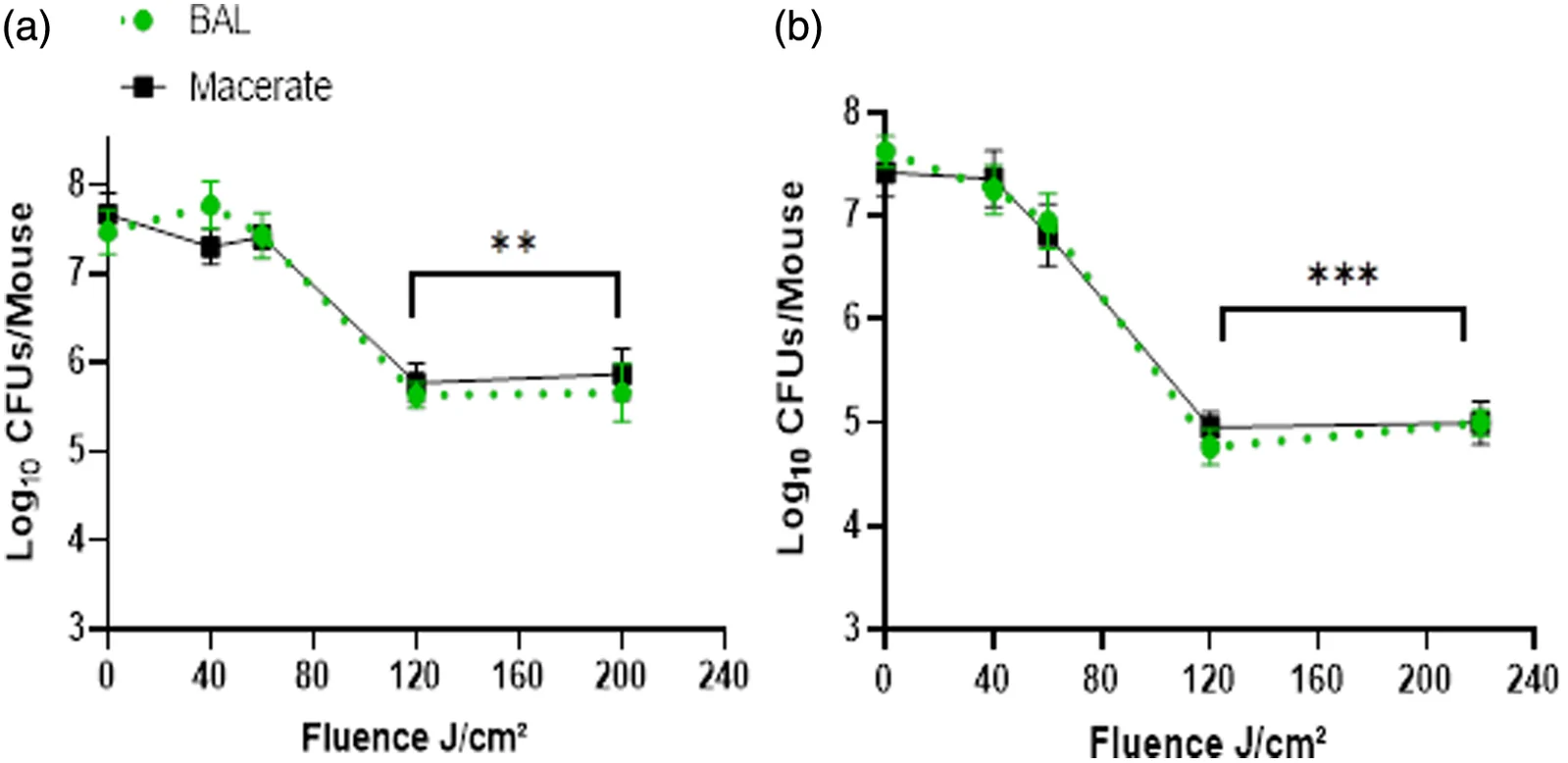
Recovered bacterial load (CFU/mouse) after a single aPDT treatment using instillation (A) or nebulization delivery (B) at different delivered fluences. ***p<0.01 and **p<0.001 versus the infected untreated control group. n=5.

The animals received the Gantrez-ICG formulation via intratracheal instillation or nebulization. The irradiation (with fluences of 40, 60, 120, and $200\;J/{\mathrm{cm}}^{2}$) was performed using a laser-based device at 808 nm, commencing simultaneously with the drug administration and continuing for the irradiation time to achieve the desired delivered fluence. The quantification of bacteria in the bronchoalveolar lavage (BAL) and lung macerate following aPDT at different fluences is shown in Fig. 2.

The Gantrez-ICG formulation and aPDT at low fluence levels of 40 and $60\;J/{\mathrm{cm}}^{2}$ did not significantly reduce the bacteria load for any of the drug delivery methods evaluated. On the other hand, for fluences over $20\;J/{\mathrm{cm}}^{2}$, there was a significant reduction in CFU/Mouse of ∼2 and 3 log for intratracheal instillation and nebulization, respectively. No statistically significant differences were observed between the bacteria recovery methods (BAL and tissue macerate). A tendency toward better results with nebulization delivery was also observed. Moreover, it is worth noting that groups treated with ICG alone under similar conditions, also in a murine model, were previously described by Kassab, in which no significant difference was observed compared to the control group, even when multiple aPDT sessions were applied.^32^

#### 3.1.2 Dual-Treatment Response

**Fig. 3:**
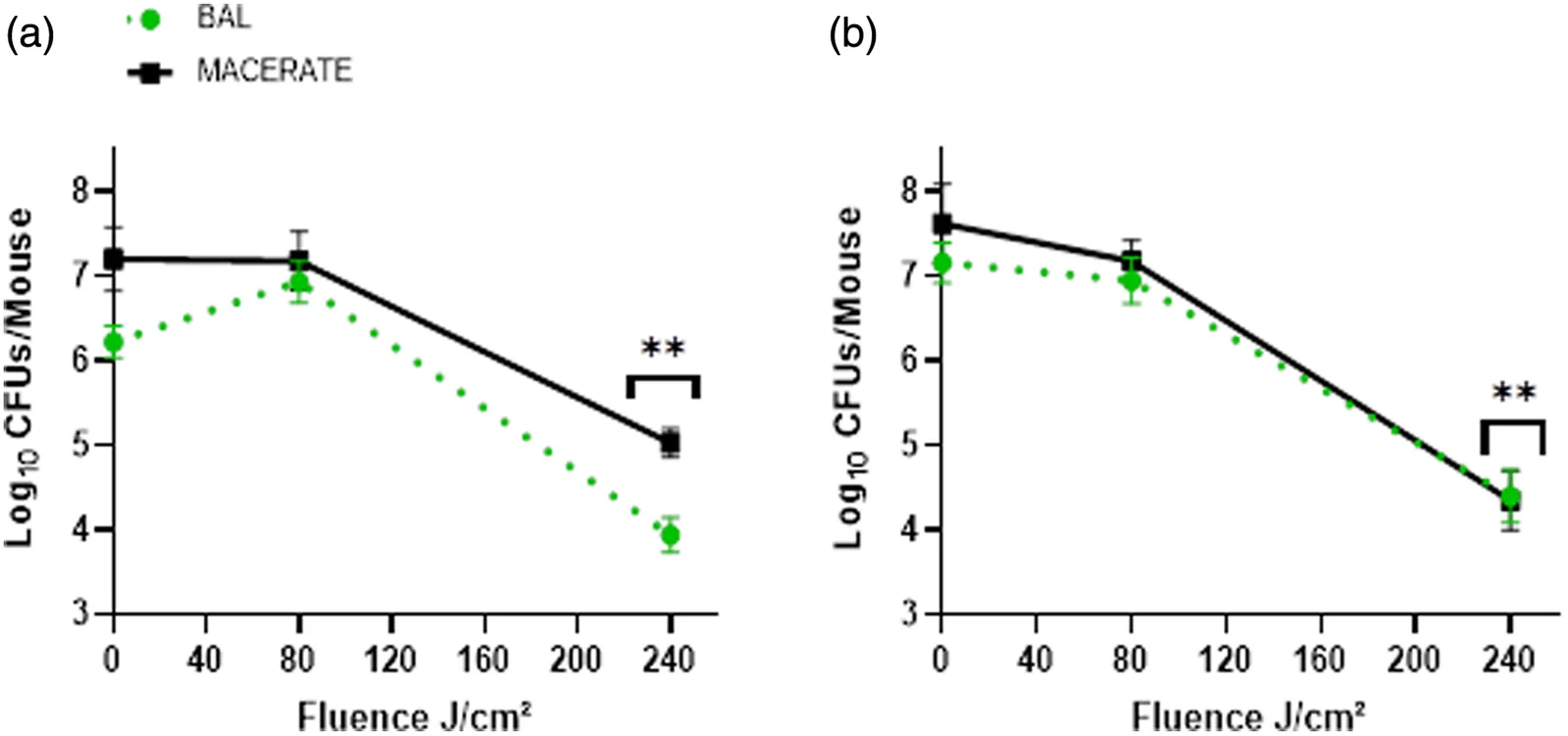
Recovered bacterial load (CFU/mouse) after dual aPDT treatment using intratracheal instillation (a) or nebulization delivery (b) at different delivered fluences. **p<0.01 versus the infected untreated control group. n=5.

To improve the efficacy of aPDT against *S. pneumoniae*, a dual-treatment protocol was tested. The Gantrez-ICG was delivered simultaneously with the light, and 1 h later, a second similar treatment was carried out. The results for cohorts treated with two treatments of $40\;J/{\mathrm{cm}}^{2}$ (total $80\;J/{\mathrm{cm}}^{2}$) and of $120\;J/{\mathrm{cm}}^{2}$ (total $240\;J/{\mathrm{cm}}^{2}$) are shown in Fig. 3.

The dual treatment did not significantly improve the treatment outcome. With bacterial reductions (CFU/Mouse) of 0.7 and ∼3 log, these results were similar to those achieved with single treatments of 40 and $120\;J/{\mathrm{cm}}^{2}$, indicating that the first PDT cycle determined the result.

### 3.2 Antimicrobial Activity of Gantrez AN-139

**Fig. 4:**
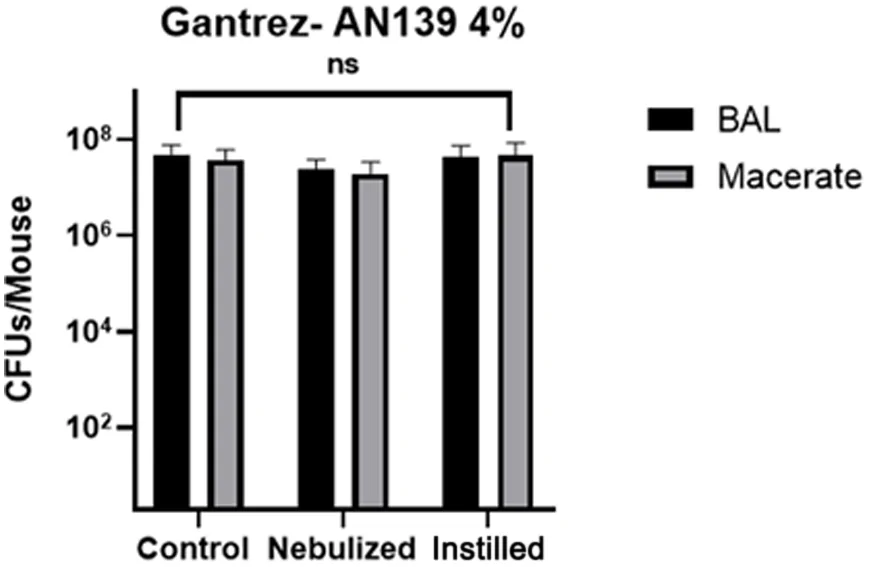
Antimicrobial effect of the Gantrez AN-139 polymer alone at a light dose of $120\;J/{\mathrm{cm}}^{2}$. ns: not significantly different versus the infected untreated control group. n=3.

Considering the intrinsic antimicrobial properties of the Gantrez polymer,^33^^,^^34^ these effects were also evaluated under light exposure. No bacterial reduction was observed in Fig. 4, confirming that the data in Figs. 2 and 3 are indeed the result of photodynamic action caused by ICG activated by light.

### 3.3 Survival Rate

**Fig. 5:**
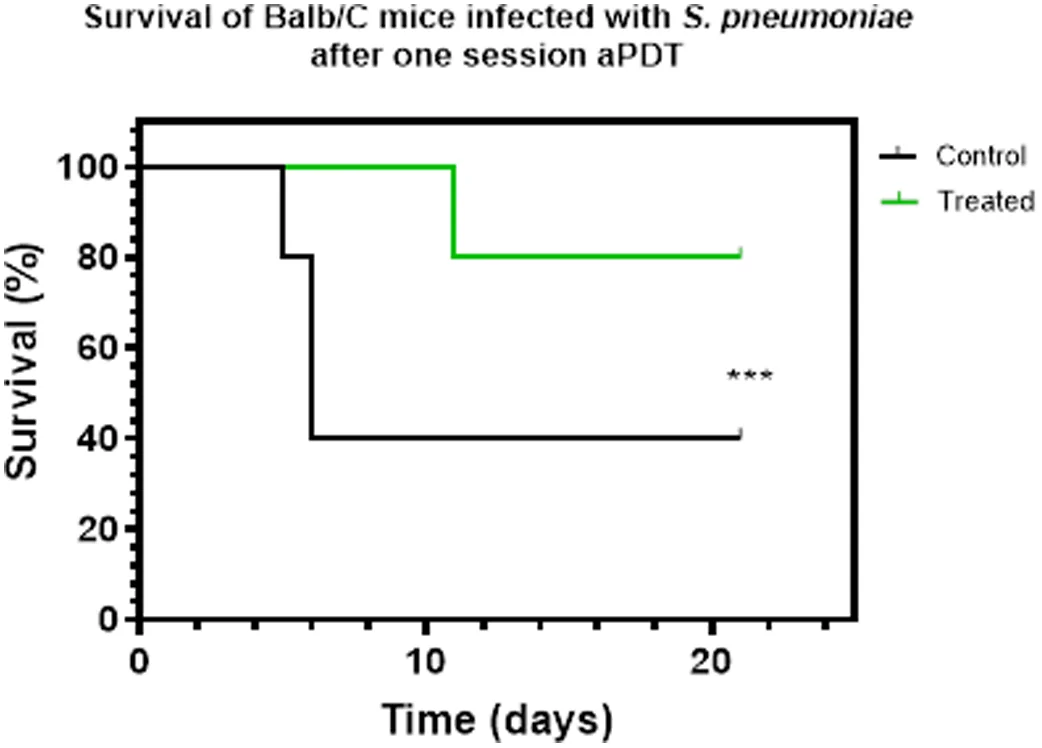
Kaplan–Meier survival curves of untreated infected control mice and mice treated with aPDT [$120\;J/{\mathrm{cm}}^{2}$ light + nebulization with 200  μmol/L ICG+4% (w/v) Gantrez] over 21 days following infection with $10^{7}$ *S. pneumoniae* cells. Treatment was performed 1 day after bacterial inoculation. In the control group, 60% of the animals (3/5) died within 6 days of infection, whereas only one animal in the aPDT group died during the 21-day observation period. *p<0.001 versus the infected untreated control group. n=5.

Based on these results, experiments were conducted to evaluate the effects of nebulized-aPDT on survival in a murine pneumonia model (n=5 per group). Animals were monitored daily for 21 days following nebulized-aPDT treatment. During the first 11 post-treatment days, the survival rate in the control group was ∼40%, compared with 80% in the aPDT group. These rates were maintained throughout the remainder of the observation period (Fig. 5).

## 4 Discussion

These findings align with a series of previously published data from our group on the efficacy of PDT for bacterial decontamination and further expand its application to external light-mediated PDT for pneumonia.^12^^,^^13^^,^^16^^–^^18^ Studies conducted by Lima et al. demonstrated that a fluence of $40\;J/{\mathrm{cm}}^{2}$, combined with 10  μM+0.2% (w/v) ICG-Gantrez, effectively inactivated bacteria ($6.31\pm 0.03\;\log_{10}\;\mathrm{CFU}/\mathrm{mL}$) and confirmed that, under these conditions, the polymer exhibits no toxicity.^19^ Based on these findings, which highlight the antimicrobial efficacy of aPDT against *S. pneumoniae*, the *in vivo* results presented in this study provide clear proof of concept for the potential translation of this technique to the treatment of pneumonia. To this end, different aPDT protocols were evaluated while maintaining the formulation ratios used *in vitro* but increasing the ICG concentration to 200  μM and the Gantrez AN-139 concentration to 4% (w/v). This adjustment was necessary because the anatomical complexity of the respiratory tract and inflammation may limit the effective delivery of the PS to the lower airways, thereby requiring higher doses to achieve therapeutic levels.^35^

In addition, Fig. 2 shows that increasing the light fluence relative to the *in vitro* experiments is also essential. When $40\;J/{\mathrm{cm}}^{2}$ was applied, no substantial bacterial reduction was observed, consistent with the expected attenuation of light by lung tissue, indicating that only a fraction of the incident light reaches the microbial target.^16^ As a result, both the nebulization and irradiation times were increased. However, no meaningful microbial inactivation occurred at $60\;J/{\mathrm{cm}}^{2}$. Significant reductions were achieved only at fluences above $120\;J/{\mathrm{cm}}^{2}$, resulting in 1.8±0.2 and 2.9±0.2 log CFU/mL reductions with intratracheal instillation and nebulization, respectively (Fig. 2), showing that the greatest reductions were obtained when ICG delivery and light dose were increased.

Regarding the instillation method for ICG delivery, it is important to consider the inherent limitations of the administration method. Although the intratracheal route enables direct delivery of the photosensitizer to the lungs, it is invasive and uncomfortable, and PS distribution is not uniform, which limits its clinical applicability.^36^ Comparative studies have shown substantial differences in pulmonary distribution between intratracheal instillation and inhalation-based administration. Multiple imaging techniques consistently demonstrate that bolus instillation results in heterogeneous deposition, predominantly within the central airways, with insufficient reach to peripheral regions.

Therefore, based on these observations and the higher antimicrobial response achieved with nebulization in the present study, this delivery method was implemented in a dual-treatment protocol to optimize therapeutic outcomes. Multiple sessions of aPDT have been widely employed in clinical studies involving different pathologies, and they often lead to improved therapeutic outcomes compared with a single application.^37^^,^^38^ Nevertheless, in this study, the second session did not confer any additional benefit (Fig. 3). There are many possible hypotheses for such a result. One possibility is that the infection and drug delivery do not fully overlap, and what was achieved and inactivated with the first procedure cannot be further treated by a second PDT cycle. In addition, the accumulation of ICG in lung tissue following repeated nebulization may have promoted aggregation and partial light attenuation due to the high local molecular density, resulting in the so-called “self-shielding” effect and consequently reducing photodynamic efficiency.^20^^,^^33^^,^^34^ Moreover, oxygen depletion induced by the first session may have further increased alveolar hypoxia, already affected by impaired gas exchange associated with pneumonia and the anesthetic regimen employed, thereby limiting the success of the second treatment, given the oxygen dependence of aPDT.^30^^,^^31^ These constraints could be addressed by adjusting treatment parameters, such as varying the interval between sessions or providing supplemental oxygen, which will be the focus of future studies.

Regarding the bacterial inactivation observed and considering the antimicrobial potential of Gantrez at specific concentrations, additional experiments were performed to ensure that the reduction was indeed attributable to aPDT.^20^^,^^24^ To this end, a control group of animals received only the polymer via nebulization and was irradiated with $120\;J/{\mathrm{cm}}^{2}$ (Fig. 4). This approach confirmed that the entire reduction in microbial burden resulted exclusively from the photodynamic action of ICG, as previously demonstrated (Figs. 2 and 3).

Finally, the survival analysis (Fig. 5), conducted over a 21-day follow-up period, provides compelling evidence of the substantial effect of aPDT with polymer-loaded ICG on overall survival. In the treated group, 80% of the animals survived, whereas survival in the control group was limited to 40%. Specifically, three of the five control animals died between days 5 and 6 post-infection, whereas only one animal in the treated group died on day 12 post-infection, corresponding to day 11 after treatment. Although complete bacterial clearance was not achieved, the reduction obtained was sufficient to support the host response, likely through immunological mechanisms, leading to infection recovery.^39^

Through different murine infection models, studies have shown that aPDT-based approaches consistently reduce inflammation while enhancing host immune responses. LD4-PDT in *K. pneumoniae* pneumonia markedly decreased cytokine levels, leukocyte infiltration, and tissue damage, improving survival. In tuberculosis models, aPDT combined with immunomodulatory nanocarriers, such as CpG-loaded PCN–PS nanoparticles or cationic photosensitizers, reduced the mycobacterial burden and attenuated granulomatous inflammation. Likewise, aPDT with hydroxyaluminum phthalocyanine decreased MDR *M. tuberculosis* load and inflammatory infiltration in multiple organs. Together, these findings demonstrate that aPDT not only inactivates pathogens but also mitigates harmful inflammatory responses and promotes immune control, reinforcing its potential as a dual antimicrobial and immunomodulatory therapy.^35^^,^^40^

An important limiting factor relates to the photodynamic performance of ICG when bound to the polymer. Preliminary data suggest that this interaction induces molecular changes that warrant further investigation. Chemical modification of ICG or incorporation of specific additives into the copolymer may further enhance its photodynamic efficiency. ICG nevertheless remains advantageous due to its near-infrared absorption, which allows deeper tissue penetration and remains a promising avenue for development.

Another concern involves the murine pneumonia model. Nasal instillation initially seeds bacteria in the upper airways before infection reaches the lower respiratory tract. Because treatment and irradiation target the lower airways, where pneumonia is established, bronchoalveolar lavage performed after therapy may displace untreated bacteria from the upper airways into the lungs, artificially elevating post-treatment microbial counts and underestimating the therapeutic effect.^41^ Even so, this model more realistically reflects the natural course of respiratory colonization.

Previous studies investigating various antimicrobial agents for the inactivation of pneumonia-causing pathogens provide a useful comparative framework for assessing the potential of aPDT.^42^^,^^43^ For instance, the analysis conducted by Kato et al., using pathogen-free female ICR Swiss mice rendered neutropenic through intraperitoneal injections of cyclophosphamide at doses of 150 and 100  mg/kg body weight, evaluated the efficacy of the antibiotics Garenoxacin (GRNX) and Levofloxacin (LVFX), administered intraperitoneally, for the treatment of mixed infections. Their results demonstrated that GRNX displayed superior antimicrobial activity compared with LVFX, achieving reductions of $2.02\;\log_{10}$ for *S. pneumoniae* and $1.12\;\log_{10}$ for *Parvimonas micra.*^43^ Although these findings highlight the effectiveness of conventional antibiotics, the aPDT outcomes obtained in the present study were even more pronounced, suggesting that both approaches could serve as complementary strategies to enhance antimicrobial efficacy.

In addition, recent evidence indicates that combining aPDT with conventional antimicrobials enhances therapeutic efficacy, particularly against multidrug-resistant pathogens.^7^^,^^44^^–^^46^ *In vitro* and *in vivo* studies show that aPDT induces structural and functional changes in bacteria, increasing antibiotic susceptibility and helping overcome resistance mechanisms. Thus, aPDT–antibiotic combinations constitute a mechanistically grounded approach to improve treatment outcomes and address the growing challenge of antimicrobial resistance.^7^^,^^44^^,^^47^^–^^49^

## 5 Conclusion

In summary, this *in vivo* study demonstrates the potential of the Gantrez AN-139 copolymer as an effective carrier for delivering ICG to the lungs, successfully overcoming the pulmonary surfactant barrier. Nebulization combined with external near-infrared irradiation led to an almost $3\text{-}\log_{10}$ reduction in the *S. pneumoniae* burden and improved animal survival. Despite the clear limitations of the present work, the results obtained are robust, providing clear evidence of the approach’s advancement. Evidence in the literature indicates that aPDT can enhance the efficacy of conventional antibiotics, suggesting a promising avenue for therapeutic integration and representing an important direction for future investigations. Collectively, these results provide a foundation for aPDT-based strategies to improve the treatment of pulmonary infections.

## Data Availability

The data supporting the findings of this study are available from the corresponding author upon reasonable request.
